# 1,4-Dichloro­naphthalene-2,3-diol

**DOI:** 10.1107/S1600536809004310

**Published:** 2009-02-28

**Authors:** Paul D. Ahn, Roger Bishop, Donald C. Craig, Marcia L. Scudder

**Affiliations:** a School of Chemistry, University of New South Wales, Sydney, Australia 2052

## Abstract

The achiral planar (maximum deviation 0.014 Å) title compound, C_10_H_6_Cl_2_O_2_, crystallizes in the chiral space group *P*2_1_2_1_2_1_ in an arrangement incorporating conventional O—H⋯O hydrogen bonding leading to a supra­molecular chain.

## Related literature

For related structures, see: Ahn *et al.* (1995 ▶, 1996 ▶). For the synthesis, see: Zincke & Fries (1904 ▶); Ahn *et al.* (1995 ▶). For related literature, see: Coppens & Hamilton (1970 ▶).
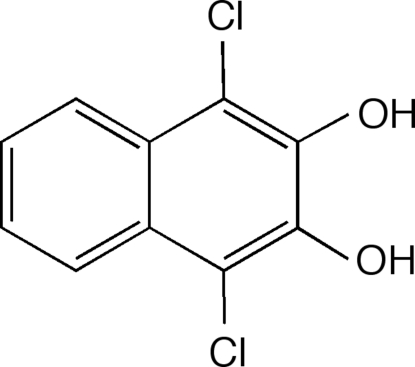


## Experimental

### 

#### Crystal data

C_10_H_6_Cl_2_O_2_
*M* *_r_* = 229.1
Orthorhombic, 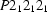

*a* = 5.0037 (4) Å
*b* = 11.589 (1) Å
*c* = 15.546 (2) Å
*V* = 901.5 (2) Å^3^
*Z* = 4
Cu *K*α radiation
μ = 6.24 mm^−1^
*T* = 294 K
0.32 × 0.09 × 0.09 mm

#### Data collection

Enraf–Nonius CAD-4 diffractometer
Absorption correction: analytical (de Meulenaer & Tompa, 1965 ▶) *T*_min_ = 0.32, *T*_max_ = 0.65
1022 measured reflections
1022 independent reflections
958 reflections with *I* > 2σ(*I*)
1 standard reflections frequency: 30 min intensity decay: none

#### Refinement

*R*[*F* ^2^ > 2σ(*F* ^2^)] = 0.022
*wR*(*F* ^2^) = 0.034
*S* = 1.38
1022 reflections
129 parameters
H-atom parameters not refined
Δρ_max_ = 0.18 e Å^−3^
Δρ_min_ = −0.17 e Å^−3^
Absolute structure: Flack (1983 ▶), no Friedel pairs
Flack parameter: 0.02 (1)

### 

Data collection: *CAD-4 Manual* (Schagen *et al.*, 1989 ▶); cell refinement: *CAD-4 Manual*; data reduction: local program; program(s) used to solve structure: *SIR92* (Altomare *et al.*, 1994 ▶); program(s) used to refine structure: *RAELS* (Rae, 2000 ▶); molecular graphics: *ORTEP-3* (Farrugia, 1997 ▶); software used to prepare material for publication: local programs.

## Supplementary Material

Crystal structure: contains datablocks global, I. DOI: 10.1107/S1600536809004310/tk2370sup1.cif

Structure factors: contains datablocks I. DOI: 10.1107/S1600536809004310/tk2370Isup2.hkl

Additional supplementary materials:  crystallographic information; 3D view; checkCIF report

## Figures and Tables

**Table 1:** Hydrogen-bond geometry (Å, °)

*D*—H⋯*A*	*D*—H	H⋯*A*	*D*⋯*A*	*D*—H⋯*A*
O1—H1*O*1⋯O1^i^	1.00	2.00	2.977 (3)	165

Symmetry code: (i) 
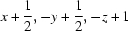
.

**Fig. 1.**
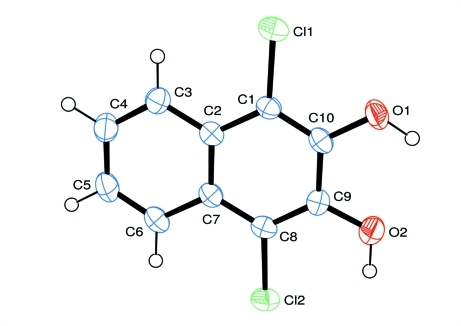
Molecular structure of (I), showing the atom labeling scheme and displacement ellipsoids at the 50% probability level.

## supplementary crystallographic information

### Comment

1,4-Dichloronaphthalene-2,3-diol forms a 2:1 inclusion compound with dioxane, the structure of which (in space group *P*2_1_/*c*) has been reported earlier (Ahn *et al.*, 1995). However, crystallization from benzene, chloroform, diethyl ether, ethanol or methanol yields solvent-free material. The crystal structures of the isomeric 1,5-dichloronaphthalene-2,6-diol, and its 1:1 inclusion compound with dioxane, have also been described (Ahn *et al.*, 1996); Fig. 1. The solvent-free title compound, (I), is planar and crystallizes such that each molecule takes part in only two hydrogen bonds (one as donor and one as acceptor), Table 1, with the same O1-hydroxy group being involved in both. This hydrogen bonding links molecules into a supramolecular chain in the *a* direction, with adjacent molecules along the chain being orthogonal. The O2—HO2 hydroxy group which does not take part in hydrogen bonding is directed towards an aromatic ring on another molecule to form an O2—HO2···π interaction with the shortest O2-H1O2···C3 and O2-H1O2···C4 distances of 2.50 and 2.58 Å, respectively. The molecules pack in a herringbone arrangement such that they are all perpendicular to the *ab* plane, maximizing opportunities for offset face-face and edge-face aromatic interactions. The former have an interplanar separation of *ca* 3.3 Å while for the latter, the C—H···C distances range up from 3.03 Å. Additionally, there are intermolecular Cl1···Cl2 interactions of 3.488 (2) Å and C—H···Cl interactions of 2.92, 3.04 and 3.09 Å and O—H···Cl of 3.05 Å.

Interestingly, this achiral molecule crystallizes in the chiral space group *P*2_1_2_1_2_1_. The 2_1_ axis along *a* accommodates the hydrogen bonding linkage while that along *b* generates the chain of molecules linked by Cl1···Cl2 interactions. The 2_1_ axis in the *c* direction leads to chains of almost coplanar molecules linked by pairs of C4—H4···Cl1 and C5—H5···Cl1 motifs.

### Experimental

1,4-Dichloronaphthalene-2,3-diol was prepared as described (Zincke & Fries, 1904; Ahn *et al.*, 1995) and X-ray quality solvent-free crystals were obtained from chloroform solution.

### Refinement

Hydrogen atoms attached to C were included at calculated positions (C—H = 1.0 Å). The hydroxy hydrogen atoms were located on a difference map, and were then fixed at a position along the OH vector with O—H = 1.0 Å. All hydrogen atoms were refined with isotropic thermal parameters equivalent to those of the atom to which they were bonded.

### Crystal data

**Table:**

C_10_H_6_Cl_2_O_2_	*F*(000) = 464.0
*M**_r_* = 229.1	*D*_x_ = 1.69 Mg m^−^^3^
Orthorhombic, *P*2_1_2_1_2_1_	Cu *K*α radiation, λ = 1.54184 Å
Hall symbol: P 2ac 2ab	Cell parameters from 10 reflections
*a* = 5.0037 (4) Å	θ = 25–30°
*b* = 11.589 (1) Å	µ = 6.24 mm^−^^1^
*c* = 15.546 (2) Å	*T* = 294 K
*V* = 901.5 (2) Å^3^	Prism, colourless
*Z* = 4	0.32 × 0.09 × 0.09 mm

### Data collection

**Table:**

Enraf–Nonius CAD-4 diffractometer	*R*_int_ = 0
ω–2θ scans	θ_max_ = 70°
Absorption correction: analytical (de Meulenaer & Tompa, 1965)	*h* = 0→6
*T*_min_ = 0.32, *T*_max_ = 0.65	*k* = 0→14
1022 measured reflections	*l* = 0→18
1022 independent reflections	1 standard reflections every 30 min
958 reflections with *I* > 2σ(*I*)	intensity decay: none

### Refinement

**Table:**

Refinement on *F*	*w* = 1/[σ^2^(*F*) + 0.0004*F*^2^]
*R*[*F*^2^ > 2σ(*F*^2^)] = 0.022	(Δ/σ)_max_ = 0.007
*wR*(*F*^2^) = 0.034	Δρ_max_ = 0.18 e Å^−^^3^
*S* = 1.38	Δρ_min_ = −0.17 e Å^−^^3^
1022 reflections	Extinction correction: (Coppens & Hamilton, 1970)
129 parameters	Extinction coefficient: 1.3 (1)
0 restraints	Absolute structure: Flack (1983), 0 Friedel pairs
H-atom parameters not refined	Flack parameter: 0.02 (1)

### Fractional atomic coordinates and isotropic or equivalent isotropic displacement parameters (Å^2^)

**Table:**

	*x*	*y*	*z*	*U*_iso_*/*U*_eq_	
Cl1	0.40159 (14)	0.04588 (5)	0.59724 (4)	0.0414 (2)	
Cl2	1.16627 (13)	0.32908 (5)	0.83291 (4)	0.0412 (2)	
O1	0.8036 (4)	0.21135 (16)	0.54315 (9)	0.0402 (4)	
O2	1.1352 (4)	0.3308 (2)	0.6420 (1)	0.0424 (5)	
C1	0.6113 (5)	0.1220 (2)	0.6655 (2)	0.0290 (5)	
C2	0.5913 (5)	0.1058 (2)	0.7565 (1)	0.0293 (5)	
C3	0.4048 (6)	0.0300 (2)	0.7943 (2)	0.0344 (5)	
C4	0.3944 (6)	0.0166 (2)	0.8827 (2)	0.0408 (6)	
C5	0.5719 (7)	0.0785 (2)	0.9350 (2)	0.0435 (6)	
C6	0.7518 (6)	0.1537 (2)	0.9010 (1)	0.0376 (6)	
C7	0.7679 (5)	0.1700 (2)	0.8102 (1)	0.0300 (5)	
C8	0.9496 (5)	0.2466 (2)	0.7716 (2)	0.0308 (5)	
C9	0.9652 (5)	0.2599 (2)	0.6839 (2)	0.0306 (5)	
C10	0.7904 (5)	0.1964 (2)	0.6298 (1)	0.0303 (5)	
H1O1	0.9726	0.2478	0.5221	0.040	
H1O2	1.2704	0.3699	0.6793	0.042	
H3	0.2790	−0.0145	0.7569	0.034	
H4	0.2609	−0.0369	0.9091	0.041	
H5	0.5660	0.0671	0.9987	0.043	
H6	0.8737	0.1979	0.9400	0.038	

### Atomic displacement parameters (Å^2^)

**Table:**

	*U*^11^	*U*^22^	*U*^33^	*U*^12^	*U*^13^	*U*^23^
Cl1	0.0418 (4)	0.0482 (3)	0.0341 (3)	−0.0060 (3)	−0.0095 (3)	−0.0051 (2)
Cl2	0.0416 (4)	0.0436 (3)	0.0385 (3)	−0.0083 (3)	−0.0052 (3)	−0.0080 (2)
O1	0.043 (1)	0.055 (1)	0.0226 (7)	−0.0045 (9)	−0.0010 (8)	0.0052 (7)
O2	0.042 (1)	0.0457 (9)	0.0392 (9)	−0.010 (1)	0.0020 (8)	0.0043 (7)
C1	0.027 (1)	0.032 (1)	0.027 (1)	0.001 (1)	−0.004 (1)	−0.0023 (8)
C2	0.031 (1)	0.030 (1)	0.027 (1)	0.003 (1)	−0.001 (1)	−0.0005 (8)
C3	0.033 (1)	0.035 (1)	0.036 (1)	0.000 (1)	0.002 (1)	0.0010 (9)
C4	0.042 (2)	0.043 (1)	0.038 (1)	−0.003 (1)	0.008 (1)	0.004 (1)
C5	0.051 (2)	0.051 (1)	0.028 (1)	0.000 (1)	0.006 (1)	0.003 (1)
C6	0.044 (1)	0.044 (1)	0.025 (1)	0.001 (1)	−0.003 (1)	−0.002 (1)
C7	0.032 (1)	0.032 (1)	0.026 (1)	0.004 (1)	0.000 (1)	−0.0005 (9)
C8	0.031 (1)	0.032 (1)	0.030 (1)	0.002 (1)	−0.004 (1)	−0.0046 (9)
C9	0.028 (1)	0.031 (1)	0.032 (1)	0.001 (1)	0.002 (1)	0.0032 (9)
C10	0.032 (1)	0.035 (1)	0.0238 (9)	0.006 (1)	−0.002 (1)	0.0002 (9)

### Geometric parameters (Å, °)

**Table:**

Cl1—C1	1.734 (2)	C6—C7	1.427 (3)
Cl2—C8	1.731 (2)	C7—C8	1.405 (3)
O1—C10	1.361 (2)	C8—C9	1.374 (3)
O2—C9	1.350 (3)	C9—C10	1.419 (3)
C1—C2	1.431 (3)	O1—H1O1	1.000
C1—C10	1.361 (3)	O2—H1O2	1.000
C2—C3	1.410 (3)	C3—H3	1.000
C2—C7	1.426 (3)	C4—H4	1.000
C3—C4	1.385 (3)	C5—H5	1.000
C4—C5	1.401 (4)	C6—H6	1.000
C5—C6	1.360 (4)		
			
Cl1—C1—C2	119.7 (2)	O2—C9—C8	125.7 (2)
Cl1—C1—C10	118.1 (2)	O2—C9—C10	114.7 (2)
C2—C1—C10	122.2 (2)	C8—C9—C10	119.6 (2)
C1—C2—C3	122.7 (2)	O1—C10—C1	121.1 (2)
C1—C2—C7	117.8 (2)	O1—C10—C9	119.4 (2)
C3—C2—C7	119.5 (2)	C1—C10—C9	119.6 (2)
C2—C3—C4	120.5 (2)	C10—O1—H1O1	114.8
C3—C4—C5	119.7 (3)	C9—O2—H1O2	115.0
C4—C5—C6	121.5 (2)	C4—C3—H3	119.7
C5—C6—C7	120.4 (2)	C2—C3—H3	119.7
C2—C7—C6	118.4 (2)	C3—C4—H4	120.2
C2—C7—C8	118.8 (2)	C5—C4—H4	120.2
C6—C7—C8	122.9 (2)	C4—C5—H5	119.3
Cl2—C8—C7	121.2 (2)	C6—C5—H5	119.3
Cl2—C8—C9	116.7 (2)	C5—C6—H6	119.7
C7—C8—C9	122.1 (2)	C7—C6—H6	119.9

### Hydrogen-bond geometry (Å, °)

**Table:**

*D*—H···*A*	*D*—H	H···*A*	*D*···*A*	*D*—H···*A*
O1—H1O1···O1^i^	1.00	2.00	2.977 (3)	165

Symmetry codes: (i) *x*+1/2, −*y*+1/2, −*z*+1.
